# Supplementary material for: Natural variation in autumn expression is the major adaptive determinant distinguishing Arabidopsis FLC haplotypes
Source: eLife. 2020 Sep 9;9:e57671. doi: 10.7554/eLife.57671 (PMC7518893; doi:10.7554/eLife.57671)
Supplement: Supplementary file 2. — Differences between the coefficients of variation of different groups (sites, features, years; 24 comparisons) [file elife-57671-supp2.docx]

| # | Comparison | Groups | Signif. Acc | Signif. NILs | Signif. combined |
| --- | --- | --- | --- | --- | --- |
| 1 | All | VIN3-independent in North Sweden 2016-7,  VIN3-independent in Norwich 2014-5,  VIN3-independent in Norwich 2016-7,  Starting levels in North Sweden 2016-7,  Starting levels in Norwich 2014-5,  Starting levels in Norwich 2016-7,  Starting levels in North Sweden 2014-5 1st planting,  Starting levels in North Sweden 2014-5 2nd planting,  Starting levels in South Sweden 2014-5,  VIN3-dependent in North Sweden 2016-7,  VIN3-dependent in Norwich 2014-5,  VIN3-dependent in Norwich 2016-7,  Combined rate in North Sweden 2014-5 1st planting,  Combined rate in North Sweden 2014-5 2nd planting,  Combined rate in South Sweden 2014-5 | * | * | * |
| 2 | Sites (features combined) | North Sweden 2016-7, Norwich 2014-5, Norwich 2016-7, North Sweden 2014-5 1st planting, North Sweden 2014-5 2nd planting, South Sweden 2014-5 |  |  |  |
| 3 | Features (2014 sites combined) | VIN3-independent, Starting levels, VIN3-dependent, Combined rate | * | * | * |
| 4 | Features (2016 sites combined) | VIN3-independent, Starting levels, VIN3-dependent | * | * | * |
| 5 | Features (sites combined) | VIN3-independent, Starting levels, VIN3-dependent, Combined rate | * | * | * |
| 6 | Features in Norwich 2014-5 | VIN3-independent, Starting levels, VIN3-dependent | * | * | * |
| 7 | Features in Norwich 2016-7 | VIN3-independent, Starting levels, VIN3-dependent |  | * | * |
| 8 | Features in Norwich | VIN3-independent, Starting levels, VIN3-dependent | * | * | * |
| 9 | VIN3-independent and Starting levels in Norwich | VIN3-independent, Starting levels |  | * | * |
| 10 | VIN3-independent and VIN3-dependent in Norwich | VIN3-independent, VIN3-dependent | * | * | * |
| 11 | VIN3-dependent and Starting levels in Norwich | Starting levels, VIN3-dependent |  |  | * |
| 12 | Features in North Sweden 2014-5 1st planting | Starting levels, Combined rate | * | * | * |
| 13 | Features in North Sweden 2014-5 2nd planting | Starting levels, Combined rate | * |  | * |
| 14 | Features in South Sweden 2014-5 | Starting levels, Combined rate | * |  | * |
| 15 | Features in North Sweden 2016-7 | VIN3-independent, Starting levels, VIN3-dependent |  |  | * |
| 16 | Features in Sweden 2014-5 | Starting levels, Combined rate | * | * | * |
| 17 | Features in Sweden | VIN3-independent, Starting levels, VIN3-dependent, Combined rate | * | * | * |
| 18 | Starting levels | Starting levels in North Sweden 2016-7,  Starting levels in Norwich 2014-5,  Starting levels in Norwich 2016-7,  Starting levels in North Sweden 2014-5 1st planting,  Starting levels in North Sweden 2014-5 2nd planting,  Starting levels in South Sweden 2014-5 |  |  |  |
| 19 | Shutdown rates | VIN3-independent in North Sweden 2016-7,  VIN3-independent in Norwich 2014-5,  VIN3-independent in Norwich 2016-7,  VIN3-dependent in North Sweden 2016-7,  VIN3-dependent in Norwich 2014-5,  VIN3-dependent in Norwich 2016-7,  Combined rate in North Sweden 2014-5 1st planting,  Combined rate in North Sweden 2014-5 2nd planting,  Combined rate in South Sweden 2014-5 |  | * | * |
| 20 | Combined shutdown rates (Sweden 2014-5) | Combined rate in North Sweden 2014-5 1st planting,  Combined rate in North Sweden 2014-5 2nd planting,  Combined rate in South Sweden 2014-5 |  |  |  |
| 21 | VIN3-independent rates | VIN3-independent in North Sweden 2016-7,  VIN3-independent in Norwich 2014-5,  VIN3-independent in Norwich 2016-7 |  |  |  |
| 22 | VIN3-dependent rates | VIN3-dependent in North Sweden 2016-7,  VIN3-dependent in Norwich 2014-5,  VIN3-dependent in Norwich 2016-7 |  |  |  |
| 23 | Shutdown rates (sites combined) | VIN3-independent, VIN3-dependent, Combined rate | * | * | * |
| 24 | VIN3-independent and Starting levels (sites combined) | VIN3-independent, Starting levels | * | * | * |
