# Supplementary material for: Natural variation in autumn expression is the major adaptive determinant distinguishing Arabidopsis FLC haplotypes
Source: eLife. 2020 Sep 9;9:e57671. doi: 10.7554/eLife.57671 (PMC7518893; doi:10.7554/eLife.57671)
Supplement: Supplementary file 4. [file elife-57671-supp4.docx]

#### Supplementary File 4, Supplementary Table 1. Sources of previously published mutants and transgenics.

| Genotype | Details | Norwich 2014 | South Sweden 2014 | North Sweden 2014 | Norwich 2016 | South Sweden 2016 | North Sweden 2016 | Source |  |
| --- | --- | --- | --- | --- | --- | --- | --- | --- | --- |
| Col *FRI* | Col-0 with *FRI* from San Feliu introgressed | X | X | X | X | X | X | Lee and Amasino (1995) |  |
| ***Accessions*** | | | | | | | | | |
| Col-0 |  | X | X | X | X | X | X | Shindo et al. (2005) |  |
| Bro1-6 |  | X | X | X | X | X | X | Long et al. (2013) |  |
| Edi-0 |  | X | X | X | X | X | X | Shindo et al. (2005) |  |
| Löv-1 |  | X | X | X | X | X | X | Shindo et al. (2005) |  |
| Ull2-5 |  | X | X | X | X | X | X | Shindo et al. (2005) |  |
| Var2-6 |  | X | X | X | X | X | X | Shindo et al. (2005) |  |
| ***Near Isogenic Lines*** | | | | | | | | | |
| Bro NIL | *FLC* from Bro1-6 backcrossed to Col *FRI* background six times. |  |  |  | X |  |  | This paper |  |
| Edi NIL | *FLC* from Edi-0 backcrossed to Col *FRI* background six times. | X | X | X | X | X | X | This paper |  |
| Löv NIL1 |  | X | X | X | X | X | X | Duncan et al. (2015) |  |
| Löv NIL2 |  | X | X | X | X | X | X | Duncan et al. (2015) |  |
| Ull NIL | *FLC* from Ull2-5 backcrossed to Col *FRI* background six times. |  |  |  | X | X | X | This paper, derived from Strange et al. (2011) |  |
| Var NIL |  | X |  |  | X | X | X | Li et al. (2015) |  |
| ***Mutants*** | | | | | | | | | |
| *vin3-1 FRI* |  |  |  |  | X | X | X | Sung & Amasino (2004) |  |
| *vin3-4 FRI* |  | X |  |  | X |  |  | Bond et al. (2009b) |  |
| *vrn1-4 FRI* |  | X |  |  |  |  |  | Sung & Amasino (2004) |  |
| *vrn2-1 FRI* |  | X |  |  | X |  |  | Yang et al. (2017) |  |
| *vrn5-8 FRI* |  |  |  |  | X |  |  | Greb et al. (2007) |  |
| *ndx1-1 FRI* |  | X |  |  | X |  |  | Sun et al. (2013) |  |
| *fca-9* |  | X |  |  |  |  |  | Liu et al. (2007) |  |
| *fld-4* |  | X |  |  |  |  |  | Liu et al. (2007) |  |
| *fve-3* |  | X |  |  |  |  |  | Ausín et al. (2004) |  |
| *fca-9 FRI* | Cross Col *FRI* and lines reported above |  |  |  | X |  |  | This paper |  |
| *fld-4 FRI* | Cross Col *FRI* and lines reported above |  |  |  | X |  |  | This paper |  |
| *fve-3 FRI* | Cross Col *FRI* and lines reported above |  |  |  | X |  |  | This paper |  |
| *val1-2 FRI* |  |  |  |  | X |  |  | Qüesta et al. (2016) |  |
| *sdg8 FRI* |  |  |  |  | X |  |  | Yang et al. (2014) |  |
| *lhp1-3 FRI* |  |  |  |  | X |  |  | Mylne et al. (2006) |  |
