# Supplementary material for: Natural variation in autumn expression is the major adaptive determinant distinguishing Arabidopsis FLC haplotypes
Source: eLife. 2020 Sep 9;9:e57671. doi: 10.7554/eLife.57671 (PMC7518893; doi:10.7554/eLife.57671)
Supplement: Supplementary file 5. [file elife-57671-supp5.docx]

#### Supplementary File 5, Supplementary Table 2. Primers used for PCR.

| Target | Primer name | Sequence 5’-3’ | Used for RT reaction |
| --- | --- | --- | --- |
| At5g25760  control | UBC_qPCR_F | CTGCGACTCAGGGAATCTTCTAA |  |
|  | UBC_qPCR_R | TTGTGCCATTGAATTGAACCC | Y |
| At1g13320  control | PP2A QPCR F2 | ACTGCATCTAAAGACAGAGTTCC |  |
|  | PP2A QPCR R2 | CCAAGCATGGCCGTATCATGT | Y |
| At5g10140 | FLC_4265_F (spliced sense) | AGCCAAGAAGACCGAACTCA |  |
|  | FLC_5683_R (spliced sense) | TTTGTCCAGCAGGTGACATC | Y |
| At5g10140 | FLC_3966_F (unspliced sense) | CGCAATTTTCATAGCCCTTG |  |
|  | FLC_4135_R (unspliced sense) | CTTTGTAATCAAAGGTGGAGAGC |  |
|  | FLC unspliced RT (4029) | TGACATTTGATCCCACAAGC | Y |
| At5g57380 | VIN3 qPCR 1 F | TGCTTGTGGATCGTCTTGTCA |  |
|  | VIN3 qPCR 1 R | TTCTCCAGCATCCGAGCAAG | Y |
| At2g46830 | JF118-CCA1-F | CTGTGTCTGACGAGGGTCGAA |  |
|  | JF119-CCA1-R | ATATGTAAAACTTTGCGGCAATACCT | Y |
